# New Appliances and Things Medical

**Published:** 1896-07-18

**Authors:** 


### NEW APPLIANCES AND THINGS MEDICAL.

[We shall be glad to receive, at our Office, 428, Strand, London, W.C., from the manufacturers, specimens of all new preparations and appliances, which may be brought out from time to time.]

### ELASTIC KNEE CAPS, &c.

(Surgical Appliance Manufacturing Company, 138, Sherwood Street, Nottingham.)

A catalogue of the appliances manufactured by the above company, and an example of their goods have recently been forwarded to us. In comparing the prices quoted by this firm with those ordinarily exacted, one is most favourably impressed, abdominal belts ranging from 4s. 6d. upwards, and elastic stockings from 5s. 6d. The methods adopted by this firm for ensuring accurate fit, and the directions for obtaining exact measurement are painstaking and to the point. Their elastic knee caps are particularly well made, consisting of the best silk and elastic carefully shaped and well put together, and costing only 3s. 6d.

### EXCELSIOR WHISKY.

(Margrave Brothers, Llanelly and Glasgow.)

A sample of this Scotch whisky has been forwarded to us, and we find it remarkably mellow, of most agreeable blend, and evidently well matured, a combination of qualities which eminently fits it for use among invalids, and especially among the rheumatic and podagric contingent, who are often debarred from other forms of stimulant.

